# The Microbiome Revolution: New Insights for Personalized Medicine

**DOI:** 10.3390/jpm12091520

**Published:** 2022-09-16

**Authors:** Lucrezia Laterza, Irene Mignini

**Affiliations:** CEMAD Digestive Diseases Center, Fondazione Policlinico Universitario “A. Gemelli” IRCCS, Università Cattolica del Sacro Cuore, 00168 Rome, Italy

The availability of new culture-independent techniques to study microbes led to the explosion of the gut microbiota revolution in recent decades. Thanks to the information deriving from 16-RNA and metagenomics, we have begun to gain insight into a previously unexplored organ: the microbiome. Thus, we changed our ontological perspective of the human body, considering it as the result of the interaction between eukaryotic cells—the body as traditionally intended—and prokaryotic cells, consisting in the multiple microorganisms living in different body niches, which constitute the “mammalian holobiont” [1]. In fact, even if the gut microbiome is the first and most studied one, multiple microbiomes have been evaluated. Previously considered sterile districts have been questioned and new possible unexpected niches have been postulated. Beyond the biological interest, the knowledge on the microbiome offers a unique opportunity for personalized medicine as it constitutes the variable part of our genome, contributing to more than 90% of the variability among the individuals, whereas our traditionally intended genome shows a more than 90% identity among individuals [2]. Furthermore, beyond the mere characterization of microorganisms constituting the microbiome, thanks to proteomics and metabolomics, we can characterize the possible function that they have in physiologic and pathologic processes [3].

Considering these characteristics, the study of the microbiome will provide new opportunities to understand the pathogenesis of many non-communicable diseases, and it may be used for diagnosis, staging and tailoring different therapies on individual specific characteristics. Aiming at exploring the potential applications of microbiome in the personalization of medicine, we propose this Special Issue to collect new original data on the study of the microbiome and to offer the reader the main novelties in the landscape of the microbiome in the perspective of personalized medicine.

In such a context, the gut microbiome plays an undoubtedly pivotal role, not only in gastrointestinal but also extra-gastrointestinal disorders.

In gastrointestinal diseases, the contribution of the gut microbiota in the pathophysiology of both organic and functional disorders is now largely recognized. The review by Piccioni et al. underlined how dysbiosis, causing imbalance between pro-inflammatory and anti-inflammatory bacterial species, can trigger mucosal and peri-visceral inflammation leading to acute diverticulitis, and hypothesized a possible therapeutic role of probiotics in this setting, which is still to be confirmed by further evidence [4]. Grigor’eva et al. analysed fecal microbiota in patients affected by gallbladder stones and described how cholecystectomy can modify bacterial abundance [5]. Moreover, Barandouzi et al. showed that the microbiome composition was different in patients with irritable bowel syndrome and healthy controls and revealed the association between bacterial diversity and the consumption of specific foods such as caffeine, thus highlighting the interaction between microbiota and the environment [6]. This relationship has also been confirmed by data concerning non-gastrointestinal settings, as it is described in the study by Lin et al., which focused on a population of patients undergoing hemodialysis and analysed the impact of beta-blockers on the gut microbiome [7].

The potential link between the gut microbiome and apparently disconnected diseases has been investigated in multiple different contexts. Some districts, notably the urinary tract, can be more easily colonized by gut bacteria due to their intestinal contiguity. On this basis, our group examined how gut microbiome and intestinal barrier disruption can impact on the pathogenesis of urinary tract infections, particularly recurrent cystitis [8]. Nonetheless, the role of the gut microbiome is not limited to contiguous segments. As Cornejo-Pareja et al. showed in their pilot study about autoimmune-based thyroid diseases, gut microbiome alterations may contribute to the impairment of the immune system and subsequent onset of autoimmune disorders [9]. Furthermore, the pilot study by Chernevskaya et al. underlined the possible role of gut microbiota in identifying patients with a high risk of postoperative complications after cardiac surgery [10]. Similarly, Yamamura et al. demonstrated that the characteristics of lipid and energy metabolism of gut microbiota were able to predict a response to probiotic therapy in patients with schizophrenia [11].

However, the gut microbiome is not the unique target for diagnosis and therapy, and growing interest is rising around other microbiomes. In this context, it is worth noting that recent findings have also revealed the presence of bacterial species in some niches which were traditionally considered sterile, questioning an old paradigm and providing new potential therapeutic targets.

Indeed, Bellando-Randone et al. summarized current evidence about the contribution of oral microbiomes in the pathogenesis of different rheumatic diseases and propose to apply artificial intelligence, especially machine learning, to understand the link between oral microbiota and rheumatic diseases [12]. Chuang et al. observed that the tonsil microbiota composition was associated with chronic tonsillitis and oxygen desaturation in children affected by obstructive sleep apnea syndrome [13]. Moreover, in two different studies, Higuchi et al. evaluated the microbiome of two previously unexplored districts. They analysed in particular mesothelioma-specific [14] and thymoma-specific [15] microbiota, using resected or biopsied mesothelioma samples and resected thymoma samples, respectively, suggesting a possible microbial role in cancerogenesis. Similarly, based on emerging evidence that the uterus is not sterile, Boutriq et al. presented in their review the most recent data concerning the potential role of the endometrial microbiome in endometrial cancer and its possible interplay with the gut microbiome [16].

From diagnosis, research is now moving towards therapeutic objectives. Microbiome modulation through pre-biotics, pro-biotics and non-absorbable oral antibiotics (e.g., rifaximin) is an already a diffuse practice, especially but not only in gastrointestinal disorders, but further investigation is needed to identify more personalized treatments [17,18]. Di Leo et al. administered rifaximin to mice models for IgA nephropathy and observed symptom improvement, suggesting an unexplored therapeutic direction [19]. Fecal microbiota transplantion (FMT) usefulness in re-establishing intestinal eubiosis is widely recognized and recommended for specific indications, notably recurrent *Clostridioides difficile* colitis, but its role seems not to be limited to this setting, and it has been explored in multiple diseases, as Pession et al. showed in their review addressing the role of FMT in allogenic hematopoietic stem-cell recipients [20]. Not only *Clostridioides* infections, but also the treatment of gut graft-versus-host disease and prevention of gut dysbiosis or decolonization from antibiotic-resistant bacteria could be the main applications. Furthermore, in order to promote research and novel therapeutic strategies, Liang et al. developed a rabbit model of cystic fibrosis that, as well as cystic fibrosis human patients, was characterized by a dysbiotic microbiota compared to wild-type rabbits [21].

Giant steps have been made so far on microbiome investigation from the perspective of personalized medicine. However, certain aspects remain unclear. For example, as Basson et al. highlighted, poor study reproducibility is one of the current concerns [22]. They speculated on the possible causes of study irreproducibility and proposed some solutions, thus indicating lights and shadows on our current knowledge on this complex and challenging topic that the microbiome represents. As this Special Issue pointed out, actual data are very promising, but research should keep on exploring the various unsolved issues to perfect the use of microbiome data for the personalization of medicine.

## Data Availability

Not applicable.
